# Lessons learned from a study based on the AHP method for the assessment of sustainability in neighborhoods

**DOI:** 10.1016/j.mex.2023.102440

**Published:** 2023-10-14

**Authors:** Vanessa Guillén-Mena, Felipe Quesada-Molina, Sebastian Astudillo-Cordero, Manuel Lema, Jessica Ortiz-Fernández

**Affiliations:** a Energy Engineering Department, Faculty of Engineering of Bilbao, University of the Basque Country UPV/EHU, Pza. Ingeniero Torres Quevedo 1, Bilbao 48013, Spain; b ECOS Research Group, Faculty of Architecture and Urbanism, University of Cuenca, Cuenca 010203, Ecuador; c Interdisciplinary Space and Population Department, University of Cuenca, Cuenca 010203, Ecuador

**Keywords:** Analytic hierarchy process, Multicriteria decision making, Consensus method, Sustainable urban development, AHP for indicator prioritization

## Abstract

The Analytic Hierarchy Process (AHP) is a method that allows complex decisions to be made from impartiality, making it suitable for reaching a consensus among experts seeking to solve a problem. This method has been successfully applied in other investigations, and its use has been extended to several disciplines. This technical paper presents the lessons learned from a study that relied on the AHP method to determine priority aspects for sustainable neighborhoods. The research is developed in three replicable phases. In each of them, aspects that are recommended to be considered are detailed, for example, in the formulation of the hierarchical structure, selection of experts, expert survey design, and information processing for the determination of weights and levels of importance.

• The utilization of software to apply the AHP method can help researchers to optimize time and resources.
• Social networks proved to be more effective than conventional methods for identifying and contacting experts.
• Subjective sustainability issues can be prioritized by expert consensus.

## Graphical abstract

**Figure:**
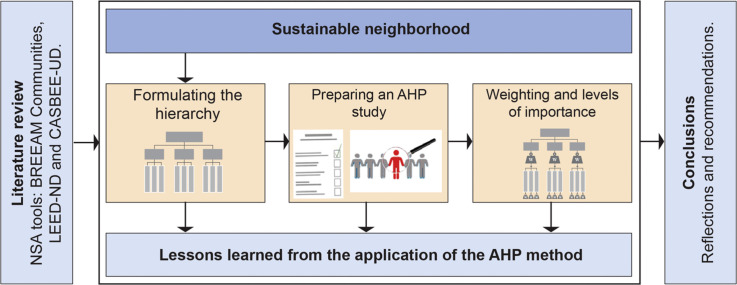

Specifications table

**Table:** 

Subject area:	Environmental Science
More specific subject area:	Sustainable urban indicators
Name of your method:	AHP for indicator prioritization
Name and reference of original method:	AHP [1]. T. L. Saaty, A scaling method for priorities in hierarchical structures. Journal of mathematical psychology, 15(3), 234–281, 1977. [2]. R. W. Saaty, The analytic hierarchy process-what it is and how it is used. Mathematical Modelling, 9(3–5), 161–176, 1987. [3]. T. L. Saaty, The modern science of multicriteria decision making and its practical applications: The AHP/ANP approach. Operations Research, 61(5), 1101–1118, 2013.
Resource availability:	N.A.

## Method details and co-submitted research

### Overview of the AHP method

The Analytical Hierarchy Method (AHP) proposed by Professor Thomas L. Saaty in 1977 [1] is a multi-criteria decision-making tool that has been used in various fields of economics, politics, and engineering to deal with complex decisions [2]. In a broader sense, it enables the transformation of a multidimensional problem (multicriteria) into a problem with a one-dimensional scale (priority scale). For this purpose, a hierarchical structure is constructed, representing all relevant aspects such as actors, scenarios, factors, elements, and interdependencies. The decision problem involves selecting the alternative that best contributes to the accomplishment of the higher-level goal. At its top level is the objective to be achieved (level 1), and at its base, the possible alternatives to be evaluated, which can be distributed into criteria (second level) and sub-criteria (third level). Comparative judgments are required for pairwise comparison of criteria and sub-criteria through a numerical scale to derive weights and define priorities. The AHP methodology has the following characteristics [[3], [4]]:

• It is a technique that allows problem-solving incorporating tangible and intangible aspects into the model, as well as subjectivism and uncertainty inherent in the decision-making process.
• It is a general theory of measurement that is applied to the influence between alternatives concerning a criterion or attribute.
• Generally, the information obtained is redundant and may be inconsistent, processed through the comparison matrices containing the judgments.
• Once the contributions to the higher elements of the hierarchy have been evaluated, the contributions of each alternative to the overall objective are calculated through additive aggregation.

The application of this method in various areas has been very convenient to pursue different purposes. Specifically, in the fields of engineering and environmental sciences, the AHP method has been employed in numerous research studies. For instance, Leccese et al. [5] introduced the use of AHP to assess the relevance of each environmental factor and improve Indoor Environment Quality (IEQ) within school buildings. Other research has focused on analyzing specific environmental factors, such as acoustic or lighting comfort, for enhancing speech intelligibility and assessing lighting quality [[6], [7]]. Likewise, the AHP has been used to develop an energy efficiency rating system for buildings [[8], [9]], to evaluate their sustainability [[4], [10], [11]], or to determine energy efficiency strategies in lighting [12]. AHP was also applied to evaluate bioenergy developments and to select the most appropriate renewable energy package [[13], [14]]. At the urban level, the AHP method has been valuable for the development of neighborhood sustainability assessment tools and prioritizing indicators according to local needs [[15], [16], [17]]. In other words, the AHP method is widely employed to adapt international tools to suit local contexts. For instance, Ameen and Mourshed [16] identified urban indicators relevant to the Iraq context and assigned weights using the AHP. Additional examples of AHP application are evident in the research developed for Qatar (Qatar Sustainability Assessment System - Neighborhood Development) and Iran (Sustainable Urban Development) [[15], [17]].

### Co-submitted research

The technical document is based on the application of the AHP method to determine which sustainability indicators should be evaluated for a locality in Ecuador (the city of Cuenca). The application of sustainable indicators in urban development supports nature-friendly practices and contributes to improving people's quality of life [18]. Thus, the high amounts of raw materials and energy resources consumed by cities, with their consequent gas emissions and solid waste, can be reduced or avoided. For the application of indicators, it is necessary to analyze from a sustainability perspective, all aspects surrounding a particular city and its interactions to identify the key issues to be addressed. This is crucial because urban priorities differ between cities, regions, and countries [[16], [19]], especially in developing countries where social, economic, and cultural factors may demand immediate attention or hold a prominent place on local agendas [20]. In this way, considering the disparity of urban priorities between cities will allow sustainability assessment tools to be more effective in different cities and countries worldwide [21]. Nonetheless, some authors have pointed out that internationally recognized Neighborhood Sustainability Assessment (NSA) methods, such as BREEAM Community, LEED-ND, and CASBEE-UD, feature indicators, and weighting systems tailored to their countries of origin, and do not consider priorities from other regions [[22], [23]]. For this reason, the universal application of NSAs has been questioned [16], and the scientific literature has not been able to fill this gap since little is known about the robustness, usefulness, precision, validity, and viability of the definition of local priorities [[24], [25], [26]]. In this sense, this study proposes to prioritize the urban sustainability indicators, which were previously selected, to develop an NSA that responds to the needs of a city that operates with different social, economic, and environmental dynamics.

To achieve this purpose, the AHP method made it possible to place all these aspects identified as relevant in a hierarchical structure. Then, through a consultation with a group of professionals with experience in the planning and development of the locality studied, sustainable urban indicators that respond to the needs of the neighborhoods of the analyzed city were prioritized and weighted. The study was conducted between the last months of 2020 and the beginning of 2021 in a pandemic context, so virtual tools played an important role. The AHP method proved to be an appropriate technique since it allowed obtaining objective, critical, and reliable results.

### Structure and aim of this paper

The study followed a three-phase research approach: 1. Literature review for the formulation of the hierarchical structure, 2. Preparing an AHP study, and 3. Weighting and levels of importance. As part of the first phase, we focused on identifying criteria and sub-criteria based on the most relevant Neighborhood Sustainability Assessment (NSA). In the second phase, we focus on the pairwise comparison of the AHP method, especially in the development of the questionnaire and in the selection of experts. Finally, in the third phase, the comparison matrix, the calculation of weights, and the verification of consistencies are analyzed to determine the level of importance of the criteria and sub-criteria. In addition, this technical document provides practical recommendations for the research community through a discussion of the lessons learned in each phase. We also describe from our experience the aspects that we would do differently if we had to carry out a new study applying the AHP method and the advantages of using online tools to process information and reduce inconsistencies. Therefore, the main objective of this document is to provide practical recommendations for the application of the AHP multicriteria method.

## Literature review for the formulation of the hierarchical structure (Phase one)

Based on the bibliographic review, the criteria and sub-criteria were provided to solve the objective that consists of sustainable neighborhoods. This allowed for defining the hierarchical structure required for the application of the AHP method. Three NSA were chosen that are evaluation tools that provide guidelines for the inclusion of sustainability measures in the development of neighborhood projects. These systems are constituted as multi-criteria models, for which they are hierarchically structured by criteria (categories), sub-criteria (indicators), and scores. Through comparisons, it is possible to measure the relationships between the different criteria and sub-criteria, as well as to define their levels. Additionally, they incorporate a rating system that is structured based on sub-criteria, which issue scores according to the level of compliance and assign weights to the scores obtained. Finally, they issue an overall rating to the neighborhood, certifying its level of sustainable performance [23].

**Fig. 1:**
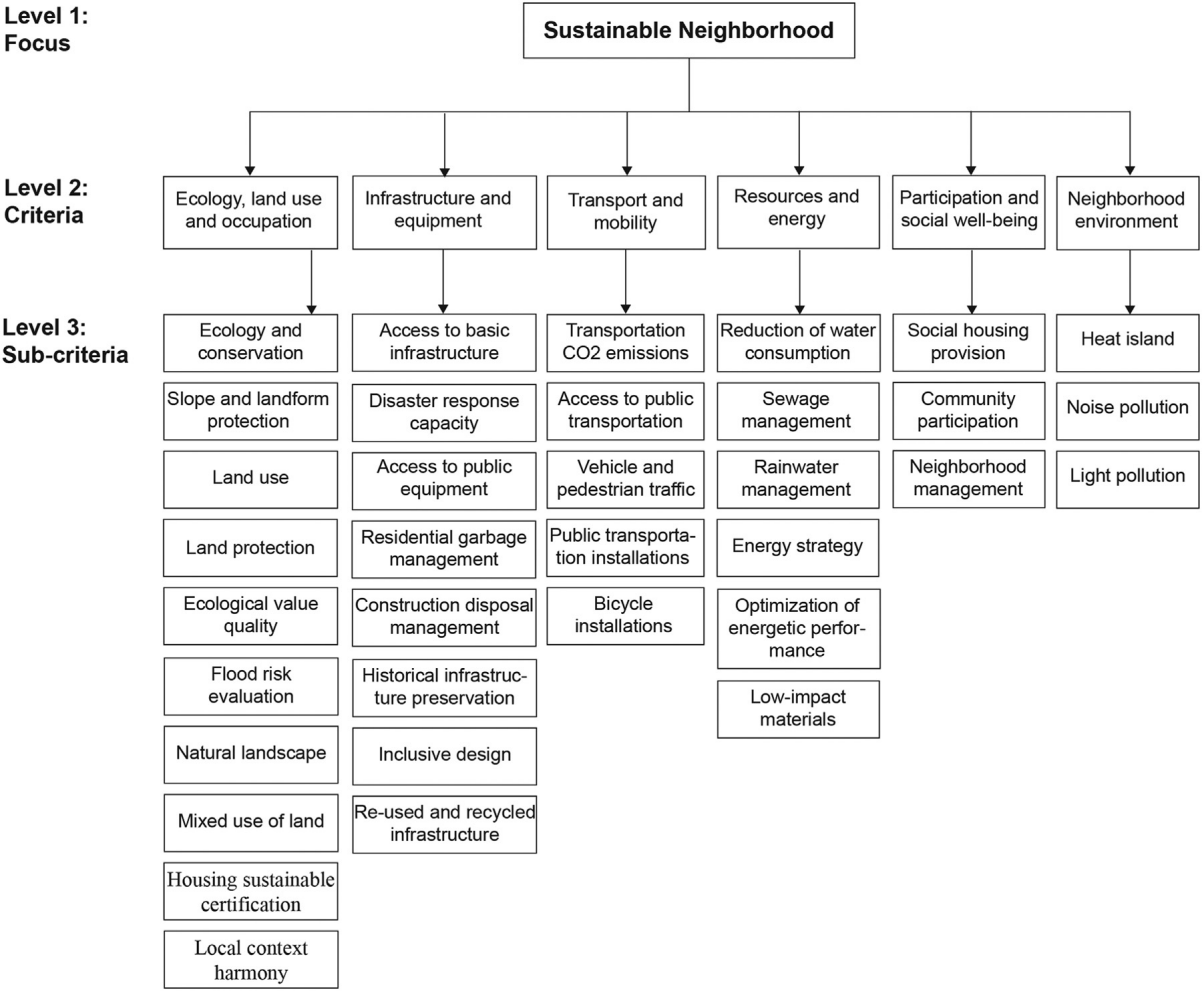
Hierarchical structure.

The three selected NSA are BREEAM Communities, LEED-ND, and CASBEE-UD. These three tools were chosen because some authors have established that they are the most internationally recognized NSA. They have the largest number of certified urban projects, and their information is freely accessible, including evaluation methodologies and scores [[15], [23], [27], [28]]. The selected NSAs have 141 sub-criteria, which were analyzed to identify those that are repeated and can be standardized. Their potential for application in the study context was also considered, depending on the availability of information needed for their evaluation. The information required for the sub-criteria was collected from different sources, including websites of institutions responsible for providing services to the local population, official diagnostic reports on required topics, interviews with municipal officials and urban promoters, perception surveys of their inhabitants, and *in-situ* information gathering. This analysis resulted in a total of 35 sub-criteria grouped into 6 criteria: 1. Ecology, land use and occupation, 2. Infrastructure and Equipment, 3. Transport and Mobility, 4. Resources and Energy, 5. Participation and Social well-being, and 6. Neighborhood Environment. The complete hierarchical structure is presented in Fig. 1.

Lessons learned from ¨Literature review for the formulation of the hierarchical structure¨

• The AHP method in its initial stage requires a bibliographic review pertinent to the object of study, which allows structuring a hierarchy through the concepts to be analyzed. Our literature review was based on the study of three of the most relevant NSA. However, it has been shown that some studies also include some additional NSA than those used in this research [29]. This may be related to the locality of study since some countries have their own assessment tools.
• Since the problem to be solved addresses various topics, it is recommended to involve local expert professionals in the homologation and selection of appropriate sub-criteria for the hierarchical structure, due their experience in addressing the analysis problem.
• It is recommended that the hierarchical structure not be extensive, since this will affect the time required to complete the questionnaire when proceeding with the AHP method. To avoid conflict over the collection of sufficient amounts of data, it is recommended that the scope of the study be clearly defined [30].
• An alternative to prevent an extensive hierarchical structure is to incorporate other methodologies, such as Fuzzy Delphi Method, with the involvement of experts to enable a more objective selection of relevant criteria for the locality [17]. This approach will result in a reduced number of criteria and sub-criteria, thus minimizing the number of judgments required when applying the AHP method. Moreover, it will enhance the agility of the method's application and reduce the challenges associated with maintaining consistent judgments.

## Preparing a AHP study (Phase two)

### Expert questionnaire survey

After defining the hierarchical structure to solve the problem, a structured survey was designed based on the criteria and sub-criteria as established by the AHP method [3]. The AHP-OS online program developed by Prof. Dr. Klaus D. Goepel was used for the survey implementation [31]. This tool serves to support decision-making, document and validate results, and even download data in Microsoft Excel easily and immediately. The explanation of this program and the user guide are published at [[32], [33]].

**Fig. 2:**
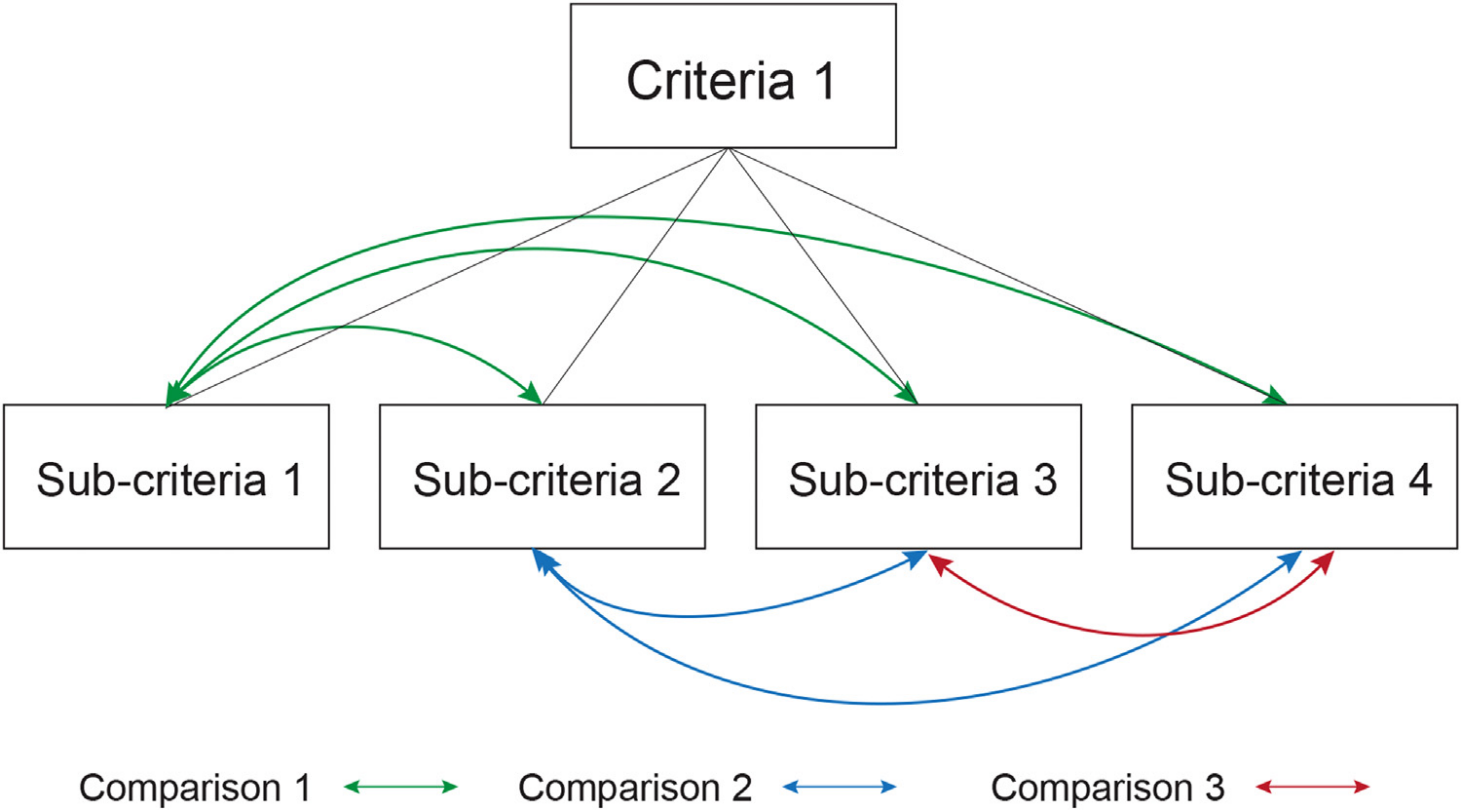
Example of pairwise comparisons between sub-criteria of a criteria.

The purpose of this survey was to determine the priority of each criteria and sub-criterion through the application of a set of forced-choice questions to collect quantitative data. The survey was structured into three levels: level 1 represents the objective, level 2 represents the criteria, and level 3 represents the sub-criteria. It was recommended to start with level 1, for which the first pairwise comparisons were made between the criteria of level 2, and so on until all comparison blocks were completed (Fig. 2). The judgment to be issued consists of selecting the most important criteria or sub-criteria from a pair and subsequently assigning a score on a scale of 1 to 9, according to Table 1.

**Table 1:** Fundamental scale of saaty.

Intensity of importance	Definition	Explanation
1	Equal importance	Two activities contribute equally to the objective.
3	Moderate importance of one over another	Experience and judgment slightly favor one activity over another.
5	Essential or strong importance	Experience and judgment strongly favor one activity over another.
7	Very strong importance	An activity is favored very strongly over another; its dominance is demonstrated in practice.
9	Extreme importance	The evidence favoring one activity over another is of the highest possible order of affirmation.
2,4,6,8	Intermediate values between the two adjacent judgments	When compromise is needed.
Reciprocals	If activity i has one of the above numbers assigned to it when compared with activity j, then j has the reciprocal value when compared with i.	

The questionnaire consisted of two parts. The first consisted of completing the respondent's data (names, surnames, and session codes). And the second part consisted of the consultation of value judgments on the criteria and sub-criteria. Before applying the survey, a virtual workshop was conducted to perform preliminary tests to verify the comprehension and reliability of the questionnaires, and to ensure that the online tool (AHP-OS) is understood by the experts. The pilot test involved four specialist professors in urban planning and two research architects who are part of the project. Along with the invitation to participate in the survey, an instruction manual and the link for the online meeting and survey were sent. During the virtual session, the project was introduced along with the hierarchical structure, outlining the objectives for each criteria and sub-criteria, as well as the methodology of the AHP method. Finally, an explanation was provided on how to fill out the survey. Based on the observations and suggestions made by the academics, the questionnaire and question wording were refined. Likewise, the comments regarding the use of the AHP-OS program were considered.

For the application of the final questionnaire, the invitations sent to the experts included information on the objective of the research project, the method used, the city for which it is applied, the duration of the survey, the use of the data, confidentiality, and contact information. Additionally, it was indicated that the survey is guided, so a link was attached to the MEET platform with an established date and time. Along with the invitation, an instruction manual and an informative poster were attached. The instruction manual explained how to access the online survey, the data to be provided, and the process for pairwise comparison. Meanwhile, the poster included the criteria and sub-criteria to be evaluated. All this information was also reviewed during the virtual session. The estimated time required to fill out the survey was one hour. Those who did not participate in this meeting were contacted to arrange individual face-to-face or virtual meetings, and they were also invited an event for Facebook Live. The survey model applied is given in the supplementary material.

Regarding AHP-OS software, it is available at https://bpmsg.com/ahp/. Logging in is necessary for generating surveys and managing information but is not required to fill it out. To create surveys, users should start by establishing criteria and then sub-criteria. The system allows for the creation of up to a total of 100 sub-criteria, with a maximum of 6000 characters. However, it is not recommended to create too many sub-criteria, especially to maintain consistent judgments. During pairwise comparisons, users have the option to calculate the Consistency Ratio (CR) for each block and make necessary corrections. In addition, results can be analyzed individually or in groups.

Lessons learned from ¨ Expert questionnaire survey¨

• The implementation of workshops for conducting the pilot survey provided valuable recommendations aimed at enhancing the survey's comprehensibility. For instance, we recognized the need to refine the wording to minimize the potential for ambiguous interpretations and to ensure the proper alignment of sub-criteria with their corresponding criteria.
• Regarding the AHP-OS program, it was found to be a user-friendly tool. The only novelty is that the program allows for survey submission, even if the percentage of inconsistencies exceeds 10 %. Therefore, these cases had to be eliminated. However, many others were corrected as the program enables the visualization of the three most inconsistent value judgments. The mathematical analysis to determine the inconsistencies can be found in Saaty [34]. For these corrections, it was crucial to have the survey guided by the research group members, as they could alert participants about this issue and explain the appropriate procedure. Thus, utilizing online programs for implementing the AHP method can provide substantial benefits to researchers, notably by reducing time, mitigating information processing errors, and enhancing the rate of survey consistency.
• Another alternative available in the AHP-OS software is the ability to correct surveys that show a ratio greater than 10 % after being saved and sent. However, this would require contacting the expert again and coordinating a survey review. In this study, the first option was chosen, and the second alternative was not pursued due to the significant number of consistent surveys reached. Nevertheless, these two options can be combined if necessary.
• One of the relevant aspects to consider is the country where an online tool is implemented since they are usually available in English, which can pose a challenge for its application in non-English-speaking countries. One of the benefits of this software is that it includes four languages: English, German, Spanish, and Portuguese. Additionally, it offers multilingual support for languages such as Chinese, Korean, and Thai, among others.
• The time required to complete the questionnaire was one hour. These improved due to the comments received during preliminary pre-tests. However, we acknowledge that one hour is still a long time, so we strongly discourage such lengthy surveys. When experts are confronted with extensive matrices containing substantial amounts of information simultaneously, they tend to become confused [35]. Several studies recommend controlling the length of questionnaires to prevent expert attrition and fatigue [[30], [36]]. To avoid conflicting with collecting sufficient amounts of data, it is recommended to clearly define the scope of the research [30]. However, the appropriate duration varies depending on the field of study, within our area (urban sustainability), we recommended not exceeding 30 min.
• Before starting the survey, we recommend providing a concise introduction that includes clear objectives of the study, so that the judgments that experts will give comply with the purpose of the research.
• The AHP-OS software has a limitation in data processing since it does not facilitate the identification of the participants' origin. This affects the division of experts by interest groups. In our study, we addressed this concern by using a supplementary form that enables us to capture this information. Consequently, we can process the results by selecting experts for specific interest groups. Another option consists of utilizing software with enhanced functionalities, such as “Expert Choice”. This software is widely used in scientific research, but its limitation is that it is a commercial paid software [37].
• An aspect identified in the software pertains to the number of characters used to name each criterion and sub-criterion. It is not advisable to exceed 45 characters, as the program does not permit viewing beyond this limit.

### Selection of experts

The number of decision makers participating in the AHP method consultation has not been determined. The characteristics of AHP participants, who make decisions, should not necessarily rely on them being experts; instead, they should possess experience and can evolve into experts through study, practice, and guidance from other decision-makers, just like in any other life activity [38]. This has resulted in the integration of varying numbers of participants in each study, with differing levels of training. Typically, these participants come from groups interested in the analysis of the problem. For instance, in a study involving four experts in municipal services, the AHP method was employed to define dimension weights for evaluating the quality of residential municipal services in the Dhaka North City Corporation area, Bangladesh [39]. In a study to determine the impacts of COVID-19 on indicators of Sustainable Development Goals in Egypt, 10 experts were chosen from the Institute of National Planning, Universiy, and the Ministry of housing [40]. In Iraq, with the objective of determining the weights for urban sustainability indicators, 20 experts were selected from various key interest groups, including academia, the government, and the private sector [16]. To determine the prioritization of wetland restoration to reduce the negative effects of droughts, a survey was applied to 25 participants, stratified into groups of wetland managers, academics, park rangers, local experts, and rural people [41]. In the context of Sub-Saharan Africa, for the selection of sustainable neighborhood evaluation indicators, a survey was administered to 50 representatives from various interest groups, including neighborhood residents, private developers, and independent professionals [[42], [43]]. In an extensive application of the AHP consultation in 31 provinces of Iran, 196 experts from academia, non-governmental organizations (NGOs), government stakeholders, and private and public sector employees were consulted to calculate the relative weights of sustainable urban development indicators for each region [17].

Finally, in the realm of developing tools for evaluating the sustainability of buildings, we can mention two investigations. The first, conducted for a developing country like Ethiopia, involved consultations with 37 experts from civil engineering, architecture, environmentalist, mechanical engineering, and construction engineering and management to determine the importance weights of the evaluation criteria [44]. The second, carried out in India, surveyed 45 experts, including civil engineers, architects, LEED professionals, and academics [45].

The diversity and absence of consensus regarding the number of participants in AHP consultations is also apparent in the utilization of AHP-OS software, which has reported its application in group consultations involving up to 320 participants in a single project [32].

In our study, before proceeding with the expert's selection for the application of the AHP method, the pre-selection criteria were defined. The panel of experts was carefully designed to admit opinions from various fields related to urban sustainability assessment. Three interest groups were established: academics, independent professionals (urban domain specialists), and public workers [[16], [40]]. The first group consisted of academics from different local universities engaged in research and scientific publications on sustainable development. The second group comprised urban planners or professionals with experience in urban project development. Lastly, the third group included officials working in departments related to city planning, including managers and decision-makers. All experts were anonymized in the results..

Potential panelists were identified by leveraging personal networks, identifying local scientific articles on urban-sustainable development, and conducting searches on Public Institutions' websites. All contacts were tabulated with information including name, profession, academic level, position, field of expertise, years of experience, and contact details. Each panelist was assigned a unique identifier, and their information was only accessible to researchers due to the presence of personal data. A total of 225 invitations were sent by email (30), office visits (5), and through social networks, such as Facebook (97), WhatsApp (83), and LinkedIn (10). Many of the invitations were not responded to or were generally rejected due to time constraints or lack of experience in applying the AHP method. In total, 89 panelists participated in the survey, leading to a response rate of 39.5 %. Among them, 2 respondents completed the survey partially, 3 surveys were not saved correctly, 9 surveys presented inconsistencies exceeding 10 %, and 75 surveys were successfully correctly.

Table 2 provides an overview of the experts who ultimately participated. From the total respondents, it was determined that 20.5 % belong to academia, 26.7 % to public workers, and 53.3 % to independent professionals. In each group, there are professionals with master's degree, and only the academia group had professionals with doctoral degree. Professional experience within the different groups varied from 2 to 25 years. The panel was also diverse in terms of gender distribution, with 39 % female participation. Considering that some areas are still predominantly male-dominated, this percentage can be seen as a positive outcome. [30].

**Table 2:** Experts overview.

Background		Option	Number	Background	Option	Number
		Academia (Universities)	15 (20.0 %)	Highest academic credentials	Undergraduate	29
Experts (*n* = 75)	Work unit	Public workers	20 (26.7 %)		Master´s	43
		Independent professionals	40 (53.3 %)		Ph.D.	3

Lessons learned from ¨selection of experts¨

• Invitations sent by email can take a long time to get a response. Multiple emails and friendly reminders can help increase the number of experts participating in the survey [30]. Another option is to leverage personal network contacts, as they have the potential to significantly expedite the process [36]. Other studies have also employed incentives as a means to boost positive responses. This is attributed to the principle of reciprocity, whereby potential respondents create a sense of obligation to comply with the survey request [46].
• It is crucial to acknowledge biases as they can occur at any part of the research process. Taking advantage of personal networks to get experts is one of the steps in which bias can be created. Table 2 revealed a predominant presence of independent professionals in the expert selection. Their heightened involvement may be attributed to personal connections with members of the research group. Adopting a strategy of selecting a diverse panel based on different ages, gender, positions, companies, etc., can help mitigate bias [30]. Another option is to use the snowball method [17].
• Although LinkedIn is a social network that provides enough information to research, select and contact panelists, it did not allow us to efficiently identify and reach out to experts due to its limited use in the study location (a city in Latin America). Instead, we found that effective social networks were Facebook and WhatsApp. Likewise, it was observed that due to the pandemic, there was a greater acceptance of participation in surveys through digital media.
• Regarding the size of the panel, there is no specific number of participants that is appropriate. the minimum requirement needed for someone to participate is to have experience in the analyzed problem. This means that it is not necessary to be an expert or have technical experience, because in AHP the optimization of a decision depends on the value system and the experience of the consistent decision-maker. Additionally, the participant is assisted by mathematical procedures that produce the best order of classification in relation to a decision framework [35].
• When it is decided to form a panel of experts, as occurs in this research, to validate its selection, criteria such as the level of preparation and years of experience of the panelists must be considered. This is because there is a positive correlation between these two aspects that enhances their expertise. Some studies take 5 or 10 years of experience as a threshold. Another way to validate the selection of experts is through self-assessment. This consists of asking them how they evaluate their expertise by answering the survey questions in which they participated [[30], [47]].
• Time of the experts is scarce, so this can affect the response rate and quality of the comments. Therefore, we recommend that you always anticipate the time that should be allocated for participating in the survey.

## Weighting and levels of importance (Phase three)

After obtaining the judgments of the experts, pairwise comparison matrices are constructed to establish the relative importance between different criteria or sub-criteria [3]. The matrices present a square structure since they are composed of integer values and their reciprocals to ensure the consistency of the comparisons (1). Its size matrix varies depending on the number of criteria and sub-criteria, and the elements on the main diagonal, when representing comparisons of an element with itself, will always have the value 1. To retrieve the weights of the experts' responses and establish priorities, the mathematical concept of the eigenvector applies through the procedure of the normalized matrix and the arithmetic mean. For this process, it is necessary to divide the individual elements of each column by the sum of column (2). Subsequently, it is required to average each row of the normalized matrix to determine the eigenvector or weight of each criteria or sub-criteria [48]. Finally, to obtain a hierarchy of global criteria, an average of the results of each expert is performed. Table 3 presents the results of the weighting of the criteria and sub-criteria in the conducted research.

(1) $$R=\left[\begin{array}{cccc} 1 & r_{12} & \cdots & r_{1n}\\ r_{21} & 1 & \cdots & r_{2n}\\ \vdots & \vdots & \ddots & \vdots \\ r_{n1} & r_{n2} & \cdots & 1 \end{array}\right]$$

(2) $$R_{Norm}=\left[\begin{array}{cccc} \frac{1}{\sum_{i=1}^{n}r_{i1}} & \frac{r_{12}}{\sum_{i=1}^{n}r_{i2}} & \cdots & \frac{r_{1n}}{\sum_{i=1}^{n}r_{in}}\\ \frac{r_{21}}{\sum_{i=1}^{n}r_{i1}} & \frac{1}{\sum_{i=1}^{n}r_{i2}} & \cdots & \frac{r_{2n}}{\sum_{i=1}^{n}r_{in}}\\ \vdots & \vdots & \ddots & \vdots \\ \frac{r_{n1}}{\sum_{i=1}^{n}r_{i1}} & \frac{r_{n2}}{\sum_{i=1}^{n}r_{i2}} & \cdots & \frac{1}{\sum_{i=1}^{n}r_{in}} \end{array}\right]$$

**Table 3:** Weighting of criteria and sub-criteria.

Criteria	Weight	Sub-criteria	Weight
C1 Ecology, land use and occupation	22.2 %	SC1.1 Ecology and conservation	13.10 %
		SC1.2 Slope and landform protection	12.05 %
		SC1.3 Land use	11.88 %
		SC1.4 Land protection	11.51 %
		SC1.5 Ecological value quality	11.12 %
		SC1.6 Flood risk evaluation	10.19 %
		SC1.7 Natural landscape	8.53 %
		SC1.8 Mixed use of land	7.82 %
		SC1.9 Housing sustainable certification	7.11 %
		SC1.10 Local context harmony	6.69 %
C2 Infrastructure and equipment	20.3 %	SC2.1 Access to basic infrastructure	20.28 %
		SC2.2 Disaster response capacity	19.44 %
		SC2.3 Access to public equipment	12.11 %
		SC2.4 Residential garbage management	11.10 %
		SC2.5 Construction disposal management	9.76 %
		SC2.6 Historical infrastructure preservation	9.65 %
		SC2.7 Inclusive design	9.40 %
		SC2.8 *Re*-used and recycled infrastructure	8.26 %
C3 Transport and mobility	17.9 %	SC3.1 Transportation CO_2_ emissions	24.16 %
		SC3.2 Access to public transportation	23.71 %
		SC3.3 Vehicle and pedestrian traffic	19.12 %
		SC3.4 Public transportation installations	16.90 %
		SC3.5 Bicycle installations	16.11 %
C4 Resources and energy	15.6 %	SC4.1 Reduction of water consumption	22.99 %
		SC4.2 Sewage management	20.52 %
		SC4.3 Rainwater management	16.21 %
		SC4.4 Energy strategy	15.01 %
		SC4.5 Optimization of energetic performance	13.96 %
		SC4.6 Low-impact materials	11.31 %
C5 Participation and social well-being	14.4 %	SC5.1 Social housing provision	44.15 %
		SC5.2 Community participation	32.38 %
		SC5.3 Neighborhood management	23.47 %
C6 Neighborhood environment	9.6 %	SC6.1 Heat island	40.01 %
		SC6.2 Noise pollution	35.63 %
		SC6.3 Light pollution	24.36 %

It is essential to consider that in order to extract reliable weights, the consistency ratio (CR) must be measured. The CR is used to assess the consistency of expert judgments. The judgment consistency can only be ensured if the result is less than 0.1 [34]; if this value is higher, it should be reviewed before prioritizing local needs. Subsequently, expert assessments that present inconsistent matrices should be eliminated. To calculate the consistency ratio, equation (3) is applied, where CI is the consistency index, and RI is the Random Index (its values depend on the size of the matrix and are provided by [34]). To obtain CI, equation (4) is applied, where λ_max_ represents the main eigenvalue, and n is the number of elements compared. In the AHP-OS software, the equation corresponding to CI is integrated into the CR calculation [32]. Therefore, the software allows you to view only the CR results.

(3) $$CR=\frac{CI}{R1}$$

(4) $$CI=\frac{\left(\lambda_{\max}-n\right)}{\left(n-1\right)}$$

Throughout the research, the consistency ratios (CR) of the obtained wights in Table 3 were adequate because they were found in the range of 0 to 0.003 (see Tables 4 and 5). Undoubtedly, using the AHP-OS software guided the experts towards appropriate evaluation. This software offers the ability to calculate the CR at the end of each block of comparisons. If the CR value exceeds 0.1, the software displays the follow notification: “Adjust highlighted judgments to improve consistency.” Consequently, while completing the survey, experts have the opportunity to review and refine their comparisons. Therefore, adequate results could be obtained in the same round in most of the surveys. Out of the 89 surveys administered, 14 had to be excluded from analysis due to various reasons: two respondents only partially completed the survey, three surveys were not saved correctly, and nine surveys exhibited a CR value exceeding 0.1. Consequently, the valid dataset comprises responses from 75 experts. Additional information about this aspect can be found in the sub-section titled “Selection of Experts”.

**Table 4:** Results of pairwise comparisons among the Criteria.

Sustainable Neighborhood Criteria (obtained consistency ratio CR = 0.002)						
	C1	C2	C3	C4	C5	C6
C1	**1**	0,63	0,98	0,66	1,75	0,72
C2	1,58	**1**	1,49	1,11	2,39	1,12
C3	1,02	0,67	**1**	0,84	1,58	0,91
C4	1,51	0,9	1,18	**1**	2,18	1,17
C5	0,57	0,42	0,63	0,46	**1**	0,62
C6	1,38	0,89	1,1	0,85	1,6	**1**

**Table 5:** Results of pairwise comparisons among the sub-criteria.

Sustainable Neighborhood Sub-Criteria										
Ecology, land use and occupation (consistency ratio CR = 0,002)										
	SC1.1	SC1.2	SC1.3	SC1.4	SC1.5	SC1.6	SC1.7	SC1.8	SC1.9	SC1.10
SC1.1	**1**	0,93	0,72	0,95	1,27	1,79	0,89	0,75	1,38	1,16
SC1.2	1,07	**1**	0,79	1,09	1,63	1,47	0,94	0,99	1,69	1,47
SC1.3	1,4	1,26	**1**	1,28	1,56	1,86	1,03	1,08	1,7	1,56
SC1.4	1,05	0,91	0,78	**1**	1,34	1,66	0,95	1,07	1,61	1,38
SC1.5	0,79	0,61	0,64	0,74	**1**	1,4	0,8	0,73	1,22	1,14
SC1.6	0,56	0,68	0,54	0,6	0,71	**1**	0,56	0,57	0,95	0,86
SC1.7	1,12	1,06	0,97	1,05	1,26	1,78	**1**	1,04	1,91	1,54
SC1.8	1,34	1,01	0,93	0,93	1,37	1,76	0,97	**1**	1,67	1,6
SC1.9	0,72	0,59	0,59	0,62	0,82	1,05	0,52	0,6	**1**	1,02
SC1.10	0,86	0,68	0,64	0,73	0,88	1,16	0,65	0,63	0,98	**1**

Infrastructure and equipment (consistency ratio CR = 0,002)										

	SC2.1	SC2.2	SC2.3	SC2.4	SC2.5	SC2.6	SC2.7	SC2.8		

SC2.1	**1**	1,83	1,29	1,26	1	0,59	0,57	1,17		
SC2.2	0,55	**1**	0,88	0,8	0,79	0,49	0,39	0,94		
SC2.3	0,77	1,14	**1**	1,02	0,88	0,51	0,49	0,99		
SC2.4	0,79	1,24	0,98	**1**	0,87	0,51	0,49	1,03		
SC2.5	1	1,26	1,14	1,15	**1**	0,55	0,54	1,22		
SC2.6	1,7	2,04	1,96	1,96	1,82	**1**	1,02	2,15		
SC2.7	1,75	2,58	2,04	2,06	1,86	0,98	**1**	2,16		
SC2.8	0,85	1,06	1,01	0,97	0,82	0,47	0,46	**1**		

Transport and mobility (consistency ratio CR = 0,003)										

	SC3.1	SC3.2	SC3.3	SC3.4	SC3.5					

SC3.1	**1**	1,68	1,16	0,94	1,35					
SC3.2	0,59	**1**	0,97	0,71	1,12					
SC3.3	0,87	1,03	**1**	0,8	1,2					
SC3.4	1,06	1,41	1,25	**1**	1,49					
SC3.5	0,74	0,89	0,83	0,67	**1**					

**Resources and energy** (consistency ratio CR = 0,001)										

	SC4.1	SC4.2	SC4.3	SC4.4	SC4.5	SC4.6				

SC4.1	**1**	0,96	1,33	0,62	0,88	0,58				
SC4.2	1,04	**1**	1,32	0,76	0,92	0,66				
SC4.3	0,75	0,75	**1**	0,55	0,7	0,52				
SC4.4	1,6	1,32	1,81	**1**	1,27	0,85				
SC4.5	1,14	1,08	1,42	0,79	**1**	0,73				
SC4.6	1,72	1,52	1,91	1,18	1,38	**1**				

Participation and social well-being (consistency ratio CR = 0,003)										

	SC5.1	SC5.2	SC5.3							

SC5.1	**1**	1,46	0,69							
SC5.2	0,69	**1**	0,56							
SC5.3	1,44	1,78	**1**							

Neighborhood environment (consistency ratio CR = 0)										

	SC6.1	SC6.2	SC6.3							

SC6.1	**1**	1,13	1,63							
SC6.2	0,89	**1**	1,47							
SC6.3	0,61	0,68	**1**							

Lessons learned from ¨Weighting and levels of importance¨

• The utilization of the online program facilitated the calculation of the weights and consistency analysis by automating these processes. Nonetheless, the researchers must master the calculations to avoid any potential misinterpretations.
• In the case of not using software, it is important to consider a broader expert base due to the potential presence of surveys with inconsistencies, as those that exceed 10 % must be eliminated. Another option is to seek mechanisms that alert the respondent to possible inconsistencies in the pairwise comparison process. A second round can also be carried out in which experts can correct judgments with inconsistencies.
• Depending on the object of study and the researchers' criteria, it is also possible to utilize the geometric mean instead of the arithmetic mean to maintain the reciprocal matrix with the expected value [[4], [48]].

## Method validation

**Fig. 3:**
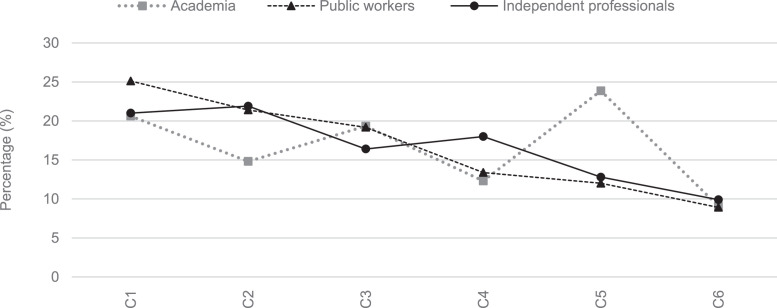
Weighting assigned to criteria by each group of experts.

**Fig. 4:**
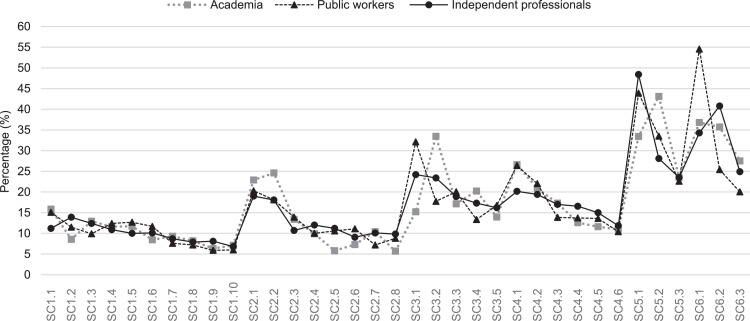
Weighting assigned to sub-criteria by each group of experts.

To validate the results obtained, an analysis of the weightings reached by groups of experts (academia, public workers, and independent professionals) is presented to determine if there is a consensus. The consensus of the group that assigns the AHP-OS tool is classified into five levels: Very low consensus when the result is below 50 % (disagreement); Low consensus between 50 % to 65 %; moderate consensus between 65 % to 75 %; high consensus between 75 % to 85 %; and very high consensus when the results are higher than 85 %. Fig. 3, Fig. 4 show that the levels of importance of the criteria and sub-criteria follow the same trend among the three groups of experts. However, there is a consensus of 54.2 %, which, although representing a low consensus, indicates an agreement.

Likewise, Tables 6 and 7 presents the weights values obtained in the criteria and sub-criteria alongside their respective percentage deviations. In most cases, the percentage deviation is close to 0 %, indicating that their comparisons are consistent. However, there are also instances negative percentage values, generally of low magnitude. These negative values account for the divergence of opinions between the groups of experts, particularly in criteria and sub-criteria such as C5 Participation and social well-being, SC3.1 Transportation CO2 emissions, SC3.2 Access to public transportation, CS5.2 Community participation and CS6.1 Heat island. Additionally, Tables 6 and 7 show the mean and standard deviation values derived from the responses provided by the three groups of experts.

**Table 6:** Statistical information from pairwise comparisons of criteria by groups of experts.

		C1	C2	C3	C4	C5	C6
Academia	Weighting	20,62 %	14,81 %	19,33 %	12,30 %	23,86 %	9,07 %
	Percentage deviation	1,63 %	4,56 %	−1,03 %	2,26 %	−7,64 %	0,22 %
	Mean	19,77 %	14,13 %	20,27 %	11,78 %	24,38 %	9,68 %
	SD	12,20 %	8,74 %	13,60 %	9,72 %	16,82 %	8,96 %
Public workers	Weighting	25,12 %	21,40 %	19,18 %	13,38 %	12,01 %	8,91 %
	Percentage deviation	−2,87 %	−2,03 %	−0,88 %	1,18 %	4,22 %	0,39 %
	Mean	24,12 %	19,49 %	19,54 %	15,20 %	12,07 %	9,58 %
	SD	12,07 %	7,71 %	12,92 %	12,03 %	8,52 %	7,90 %
Independent professionals	Weighting	21,00 %	21,90 %	16,40 %	18,00 %	12,80 %	9,90 %
	Percentage deviation	1,25 %	−2,53 %	1,90 %	−3,44 %	3,42 %	−0,61 %
	Mean	20,28 %	19,94 %	17,30 %	17,71 %	15,47 %	9,30 %
	SD	12,22 %	10,37 %	13,34 %	12,12 %	15,18 %	6,89 %

**Table 7:** Statistical information from pairwise comparisons of sub-criteria by groups of experts.

		C1									
		SC1.1	SC1.2	SC1.3	SC1.4	SC1.5	SC1.6	SC1.7	SC1.8	SC1.9	SC1.10
Academia	Weighting	15,85 %	8,57 %	12,92 %	11,59 %	11,72 %	8,44 %	9,27 %	8,20 %	6,32 %	7,12 %
	Percentage deviation	−1,80 %	2,80 %	−1,20 %	0,00 %	−0,20 %	1,60 %	−0,70 %	−0,40 %	0,50 %	−0,50 %
	Mean	13,70 %	8,88 %	14,25 %	10,97 %	11,34 %	8,70 %	8,19 %	9,11 %	7,93 %	6,94 %
	SD	6,16 %	5,90 %	10,62 %	6,75 %	6,68 %	6,50 %	4,39 %	7,80 %	9,25 %	5,12 %
Public workers	Weighting	15,10 %	11,50 %	9,90 %	12,40 %	12,70 %	11,70 %	7,60 %	7,20 %	5,90 %	6,00 %
	Percentage deviation	−1,10 %	−0,20 %	1,80 %	−0,80 %	−1,20 %	−1,60 %	0,90 %	0,60 %	0,90 %	0,60 %
	Mean	13,42 %	10,36 %	9,83 %	12,60 %	12,22 %	12,22 %	7,19 %	9,20 %	6,81 %	6,15 %
	SD	5,76 %	4,65 %	5,31 %	7,60 %	6,68 %	7,74 %	3,47 %	9,23 %	5,92 %	3,90 %
Independent professionals	Weighting	11,20 %	13,90 %	12,40 %	10,90 %	10,00 %	10,10 %	8,70 %	7,90 %	8,10 %	6,80 %
	Percentage deviation	2,90 %	−2,60 %	−0,70 %	0,70 %	1,50 %	0,00 %	−0,20 %	−0,10 %	−1,30 %	−0,20 %
	Mean	10,95 %	13,55 %	12,14 %	10,78 %	9,71 %	11,03 %	8,07 %	7,88 %	9,26 %	6,63 %
	SD	13,55 %	7,30 %	6,49 %	5,80 %	5,34 %	7,33 %	4,16 %	4,36 %	6,98 %	3,82 %


		C2								C3				
		SC2.1	SC2.2	SC2.3	SC2.4	SC2.5	SC2.6	SC2.7	SC2.8	SC3.1	SC3.2	SC3.3	SC3.4	SC3.5
Academia	Weighting	22,90 %	24,60 %	13,40 %	9,90 %	5,80 %	7,30 %	10,40 %	5,70 %	15,22 %	33,45 %	17,14 %	20,23 %	13,96 %
	Percentage deviation	−2,20 %	−4,30 %	−0,70 %	0,70 %	3,40 %	1,90 %	−1,20 %	2,40 %	8,60 %	−8,60 %	1,60 %	−3,30 %	1,70 %
	Mean	22,67 %	22,83 %	13,90 %	9,79 %	5,18 %	7,95 %	11,58 %	6,10 %	15,85 %	33,29 %	17,93 %	17,89 %	15,04 %
	SD	11,56 %	9,65 %	9,78 %	6,46 %	2,30 %	6,05 %	8,96 %	5,06 %	12,92 %	17,89 %	14,81 %	7,45 %	11,38 %
Public workers	Weighting	20,30 %	18,10 %	13,90 %	10,00 %	10,60 %	11,10 %	7,20 %	8,80 %	32,12 %	17,74 %	20,06 %	13,35 %	16,73 %
	Percentage deviation	0,40 %	2,20 %	−1,20 %	0,60 %	−1,40 %	−1,90 %	2,00 %	−0,70 %	−8,30 %	7,10 %	−1,40 %	3,60 %	−1,10 %
	Mean	20,17 %	17,70 %	15,03 %	10,96 %	10,23 %	10,58 %	6,94 %	8,39 %	31,35 %	18,44 %	18,42 %	13,09 %	18,70 %
	SD	9,74 %	10,08 %	12,24 %	8,28 %	5,91 %	6,23 %	4,44 %	4,53 %	17,55 %	11,70 %	8,76 %	8,00 %	14,92 %
Independent professionals	Weighting	19,00 %	18,08 %	10,70 %	12,01 %	11,21 %	9,07 %	10,10 %	9,83 %	24,20 %	23,40 %	18,90 %	17,30 %	16,20 %
	Percentage deviation	1,70 %	2,20 %	2,00 %	−1,40 %	−2,00 %	0,10 %	−0,90 %	−1,70 %	−0,40 %	1,50 %	−0,20 %	−0,30 %	−0,60 %
	Mean	18,67 %	17,62 %	11,67 %	11,37 %	10,34 %	10,32 %	10,27 %	9,73 %	24,95 %	22,58 %	18,03 %	16,34 %	18,10 %
	SD	10,33 %	9,85 %	9,31 %	7,38 %	5,92 %	8,60 %	8,03 %	8,21 %	15,63 %	12,09 %	12,24 %	9,38 %	15,78 %


		C4						C5			C6		
		SC4.1	SC4.2	SC4.3	SC4.4	SC4.5	SC4.6	SC5.1	SC5.2	SC5.3	SC6.1	SC6.2	SC6.3
Academia	Weighting	26,60 %	20,90 %	17,30 %	12,60 %	11,60 %	11,00 %	33,40 %	43,10 %	23,50 %	36,80 %	35,70 %	27,50 %
	Percentage deviation	−2,20 %	−0,10 %	−1,20 %	1,70 %	1,80 %	0,10 %	8,50 %	−8,20 %	−0,30 %	5,10 %	−1,70 %	−3,40 %
	Mean	24,89 %	20,46 %	16,19 %	12,89 %	12,72 %	12,85 %	36,23 %	37,92 %	25,85 %	40,81 %	33,13 %	26,06 %
	SD	13,95 %	12,27 %	7,99 %	9,52 %	10,34 %	14,78 %	28,50 %	24,54 %	26,00 %	40,81 %	16,72 %	14,09 %
Public workers	Weighting	26,38 %	22,02 %	13,86 %	13,73 %	13,61 %	10,40 %	43,90 %	33,50 %	22,60 %	54,55 %	25,41 %	20,04 %
	Percentage deviation	−2,00 %	−1,20 %	2,20 %	0,60 %	−0,20 %	0,70 %	−2,00 %	1,40 %	0,60 %	−12,70 %	8,60 %	4,10 %
	Mean	25,52 %	20,17 %	14,37 %	14,27 %	14,45 %	11,21 %	43,54 %	35,02 %	21,44 %	51,20 %	28,36 %	20,44 %
	SD	10,73 %	8,17 %	8,85 %	8,99 %	10,56 %	8,81 %	23,88 %	25,43 %	15,22 %	20,20 %	20,23 %	11,98 %
Independent professionals	Weighting	20,16 %	19,42 %	17,00 %	16,56 %	15,00 %	11,86 %	48,40 %	28,10 %	23,50 %	34,30 %	40,80 %	24,90 %
	Percentage deviation	4,20 %	1,40 %	−0,90 %	−2,30 %	−1,60 %	−0,80 %	−6,50 %	6,80 %	−0,30 %	7,60 %	−6,80 %	−0,80 %
	Mean	20,31 %	18,37 %	16,66 %	16,73 %	16,17 %	11,76 %	48,16 %	27,21 %	24,62 %	34,72 %	40,43 %	24,85 %
	SD	11,60 %	9,29 %	10,13 %	11,24 %	11,90 %	7,59 %	23,41 %	16,83 %	18,69 %	22,02 %	23,09 %	15,90 %

## Reflection and conclusion

We consider that the AHP method applies to a wide range of disciplines, and in the field of sustainability at the city level, it is a method that can assist city administrators in decision-making to impact people's quality of life, as it can address sensitive topics for society. Additionally, we believe it is a valuable method for several reasons: 1. It allowed us to break down a complex problem into manageable levels, thereby identifying local priorities in an unbiased manner, 2. We were able to include the knowledge and experience of different groups of experts that allow better support the decision-making process, 3. It is a universally applicable method, flexible and adaptable to a variety of situations and contexts, 4. It provides a visual representation of the results through the assigned weights, which facilitates communication and debate.

The limitations that we can find in the method are related to the subjectivity in expert judgments that can be influenced by personal biases and lead to low consensus or even no agreement (less than 50 % consensus). Therefore, we recommend refining the expert profile by ensuring certain conditions. For example, requiring a minimum number of years of experience and selecting experts who are knowledgeable in the specific object of study, even if they come from different disciplines. Another limitation of the method is related to the time and effort requirements, for which an accompaniment in the completion of the survey is recommended since this can positively affect optimizing time, avoiding errors, and reducing drop-out rates. However, we still consider the AHP method to be valuable in the decision-making process.

Overall, we strongly believe that the results obtained provide valuable information to guide planners and decision-makers in developing policies that enhance living conditions and promote environmentally friendly practices. Furthermore, these results contribute to prioritizing crucial aspects within a specific locality. The results are also relevant for the academic field because the study identified emerging research areas.

This technical document provided three replicable phases for applying the AHP method with the lessons learned from its application in a case study. The research was conducted in Latin America during the COVID-19 pandemic. We encourage researchers from different disciplines to utilize the AHP method for complex decision-making and to leverage online tools for survey application and information processing.

## Ethics statements

Not applicable.

## CRediT authorship contribution statement

**Vanessa Guillén-Mena:** Conceptualization, Methodology, Writing – original draft, Data curation, Formal analysis, Writing – review & editing. **Felipe Quesada-Molina:** Conceptualization, Methodology, Supervision, Formal analysis, Writing – review & editing. **Sebastian Astudillo-Cordero:** Supervision, Project administration, Funding acquisition. **Manuel Lema:** Data curation, Validation, Visualization. **Jessica Ortiz-Fernández:** Resources, Data curation, Visualization.

## Declaration of Competing Interest

The authors declare that they have no known competing financial interests or personal relationships that could have appeared to influence the work reported in this paper.

## Data Availability

Data will be made available on request.

## Appendix Supplementary materials

Image, application 1
