# Supplementary material for: Lessons learned from a study based on the AHP method for the assessment of sustainability in neighborhoods
Source: MethodsX. 2023 Oct 14;11:102440. doi: 10.1016/j.mex.2023.102440 (PMC10597795; doi:10.1016/j.mex.2023.102440)
Supplement: Supplementary file 1 [file mmc1.pdf]

AHP-OS Latest News

Pairwise Comparison AHP-OS

Evaluation of Criteria for Sustainable Neighborhood

Pairwise Comparison Sustainable Neighborhood

15 pairwise comparison(s). Please do the pairwise comparison of all criteria. When completed, click *Check Consistency* to get the priorities.

AHP Scale: 1- Equal Importance, 3- Moderate importance, 5- Strong importance, 7- Very strong importance, 9- Extreme importance (2,4,6,8 values in-between).

With respect to *Sustainable Neighborhood*, which criterion is more important, and how much more on a scale 1 to 9?

	A - wrt Sustainable Neighborhood - or B?		Equal	How much more?
1	◉ Ecology land use and occupation	○ Infrastructure and equipment	◉ 1	○2 ○3 ○4 ○5 ○6 ○7 ○8 ○9
2	◉ Ecology land use and occupation	○ Transport and mobility	◉ 1	○2 ○3 ○4 ○5 ○6 ○7 ○8 ○9
3	◉ Ecology land use and occupation	○ Resources and energy	◉ 1	○2 ○3 ○4 ○5 ○6 ○7 ○8 ○9
4	◉ Ecology land use and occupation	○ Participation and social well-being	◉ 1	○2 ○3 ○4 ○5 ○6 ○7 ○8 ○9
5	◉ Ecology land use and occupation	○ Neighborhood environment	◉ 1	○2 ○3 ○4 ○5 ○6 ○7 ○8 ○9
6	◉ Infrastructure and equipment	○ Transport and mobility	◉ 1	○2 ○3 ○4 ○5 ○6 ○7 ○8 ○9
7	◉ Infrastructure and equipment	○ Resources and energy	◉ 1	○2 ○3 ○4 ○5 ○6 ○7 ○8 ○9
8	◉ Infrastructure and equipment	○ Participation and social well-being	◉ 1	○2 ○3 ○4 ○5 ○6 ○7 ○8 ○9
9	◉ Infrastructure and equipment	○ Neighborhood environment	◉ 1	○2 ○3 ○4 ○5 ○6 ○7 ○8 ○9
10	◉ Transport and mobility	○ Resources and energy	◉ 1	○2 ○3 ○4 ○5 ○6 ○7 ○8 ○9
11	◉ Transport and mobility	○ Participation and social well-being	◉ 1	○2 ○3 ○4 ○5 ○6 ○7 ○8 ○9
12	◉ Transport and mobility	○ Neighborhood environment	◉ 1	○2 ○3 ○4 ○5 ○6 ○7 ○8 ○9
13	◉ Resources and energy	○ Participation and social well-being	◉ 1	○2 ○3 ○4 ○5 ○6 ○7 ○8 ○9
14	◉ Resources and energy	○ Neighborhood environment	◉ 1	○2 ○3 ○4 ○5 ○6 ○7 ○8 ○9
15	◉ Participation and social well-being	○ Neighborhood environment	◉ 1	○2 ○3 ○4 ○5 ○6 ○7 ○8 ○9
CR = 0% Please start pairwise comparison				
Calculate				

[AHP-OS](#) [Latest News](#)

Pairwise Comparison AHP-OS

Evaluation of Criteria for Sustainable Neighborhood

Pairwise Comparison Ecology land use and occupation

45 pairwise comparison(s). Please do the pairwise comparison of all criteria. When completed, click *Check Consistency* to get the priorities.

AHP Scale: 1- Equal Importance, 3- Moderate importance, 5- Strong importance, 7- Very strong importance, 9- Extreme importance (2,4,6,8 values in-between).

With respect to *Ecology land use and occupation*, which criterion is more important, and how much more on a scale 1 to 9?

	A - wrt Ecology land use and occupation - or B?		Equal	How much more?
1	◉ Ecology and conservation	○ Slope and landform protection	◉ 1	○2 ○3 ○4 ○5 ○6 ○7 ○8 ○9
2	◉ Ecology and conservation	○ Land use	◉ 1	○2 ○3 ○4 ○5 ○6 ○7 ○8 ○9
3	◉ Ecology and conservation	○ Land protection	◉ 1	○2 ○3 ○4 ○5 ○6 ○7 ○8 ○9
4	◉ Ecology and conservation	○ Ecological value quality	◉ 1	○2 ○3 ○4 ○5 ○6 ○7 ○8 ○9
5	◉ Ecology and conservation	○ Flood risk evaluation	◉ 1	○2 ○3 ○4 ○5 ○6 ○7 ○8 ○9
6	◉ Ecology and conservation	○ Natural landscape	◉ 1	○2 ○3 ○4 ○5 ○6 ○7 ○8 ○9
7	◉ Ecology and conservation	○ Mixed use of land	◉ 1	○2 ○3 ○4 ○5 ○6 ○7 ○8 ○9
8	◉ Ecology and conservation	○ Housing sustainable certification	◉ 1	○2 ○3 ○4 ○5 ○6 ○7 ○8 ○9
9	◉ Ecology and conservation	○ Local context harmony	◉ 1	○2 ○3 ○4 ○5 ○6 ○7 ○8 ○9
10	◉ Slope and landform protection	○ Land use	◉ 1	○2 ○3 ○4 ○5 ○6 ○7 ○8 ○9
11	◉ Slope and landform protection	○ Land protection	◉ 1	○2 ○3 ○4 ○5 ○6 ○7 ○8 ○9
12	◉ Slope and landform protection	○ Ecological value quality	◉ 1	○2 ○3 ○4 ○5 ○6 ○7 ○8 ○9
13	◉ Slope and landform protection	○ Flood risk evaluation	◉ 1	○2 ○3 ○4 ○5 ○6 ○7 ○8 ○9
14	◉ Slope and landform protection	○ Natural landscape	◉ 1	○2 ○3 ○4 ○5 ○6 ○7 ○8 ○9
15	◉ Slope and landform protection	○ Mixed use of land	◉ 1	○2 ○3 ○4 ○5 ○6 ○7 ○8 ○9
16	◉ Slope and landform protection	○ Housing sustainable certification	◉ 1	○2 ○3 ○4 ○5 ○6 ○7 ○8 ○9
17	◉ Slope and landform protection	○ Local context harmony	◉ 1	○2 ○3 ○4 ○5 ○6 ○7 ○8 ○9
18	◉ Land use	○ Land protection	◉ 1	○2 ○3 ○4 ○5 ○6 ○7 ○8 ○9
19	◉ Land use	○ Ecological value quality	◉ 1	○2 ○3 ○4 ○5 ○6 ○7 ○8 ○9
20	◉ Land use	○ Flood risk evaluation	◉ 1	○2 ○3 ○4 ○5 ○6 ○7 ○8 ○9
21	◉ Land use	○ Natural landscape	◉ 1	○2 ○3 ○4 ○5 ○6 ○7 ○8 ○9
22	◉ Land use	○ Mixed use of land	◉ 1	○2 ○3 ○4 ○5 ○6 ○7 ○8 ○9
23	◉ Land use	○ Housing sustainable certification	◉ 1	○2 ○3 ○4 ○5 ○6 ○7 ○8 ○9
24	◉ Land use	○ Local context harmony	◉ 1	○2 ○3 ○4 ○5 ○6 ○7 ○8 ○9
25	◉ Land protection	○ Ecological value quality	◉ 1	○2 ○3 ○4 ○5 ○6 ○7 ○8 ○9
26	◉ Land protection	○ Flood risk evaluation	◉ 1	○2 ○3 ○4 ○5 ○6 ○7 ○8 ○9

27	◉ Land protection	○ Natural landscape	◉ 1	○ 2 ○ 3 ○ 4 ○ 5 ○ 6 ○ 7 ○ 8 ○ 9
28	◉ Land protection	○ Mixed use of land	◉ 1	○ 2 ○ 3 ○ 4 ○ 5 ○ 6 ○ 7 ○ 8 ○ 9
29	◉ Land protection	○ Housing sustainable certification	◉ 1	○ 2 ○ 3 ○ 4 ○ 5 ○ 6 ○ 7 ○ 8 ○ 9
30	◉ Land protection	○ Local context harmony	◉ 1	○ 2 ○ 3 ○ 4 ○ 5 ○ 6 ○ 7 ○ 8 ○ 9
31	◉ Ecological value quality	○ Flood risk evaluation	◉ 1	○ 2 ○ 3 ○ 4 ○ 5 ○ 6 ○ 7 ○ 8 ○ 9
32	◉ Ecological value quality	○ Natural landscape	◉ 1	○ 2 ○ 3 ○ 4 ○ 5 ○ 6 ○ 7 ○ 8 ○ 9
33	◉ Ecological value quality	○ Mixed use of land	◉ 1	○ 2 ○ 3 ○ 4 ○ 5 ○ 6 ○ 7 ○ 8 ○ 9
34	◉ Ecological value quality	○ Housing sustainable certification	◉ 1	○ 2 ○ 3 ○ 4 ○ 5 ○ 6 ○ 7 ○ 8 ○ 9
35	◉ Ecological value quality	○ Local context harmony	◉ 1	○ 2 ○ 3 ○ 4 ○ 5 ○ 6 ○ 7 ○ 8 ○ 9
36	◉ Flood risk evaluation	○ Natural landscape	◉ 1	○ 2 ○ 3 ○ 4 ○ 5 ○ 6 ○ 7 ○ 8 ○ 9
37	◉ Flood risk evaluation	○ Mixed use of land	◉ 1	○ 2 ○ 3 ○ 4 ○ 5 ○ 6 ○ 7 ○ 8 ○ 9
38	◉ Flood risk evaluation	○ Housing sustainable certification	◉ 1	○ 2 ○ 3 ○ 4 ○ 5 ○ 6 ○ 7 ○ 8 ○ 9
39	◉ Flood risk evaluation	○ Local context harmony	◉ 1	○ 2 ○ 3 ○ 4 ○ 5 ○ 6 ○ 7 ○ 8 ○ 9
40	◉ Natural landscape	○ Mixed use of land	◉ 1	○ 2 ○ 3 ○ 4 ○ 5 ○ 6 ○ 7 ○ 8 ○ 9
41	◉ Natural landscape	○ Housing sustainable certification	◉ 1	○ 2 ○ 3 ○ 4 ○ 5 ○ 6 ○ 7 ○ 8 ○ 9
42	◉ Natural landscape	○ Local context harmony	◉ 1	○ 2 ○ 3 ○ 4 ○ 5 ○ 6 ○ 7 ○ 8 ○ 9
43	◉ Mixed use of land	○ Housing sustainable certification	◉ 1	○ 2 ○ 3 ○ 4 ○ 5 ○ 6 ○ 7 ○ 8 ○ 9
44	◉ Mixed use of land	○ Local context harmony	◉ 1	○ 2 ○ 3 ○ 4 ○ 5 ○ 6 ○ 7 ○ 8 ○ 9
45	◉ Housing sustainable certification	○ Local context harmony	◉ 1	○ 2 ○ 3 ○ 4 ○ 5 ○ 6 ○ 7 ○ 8 ○ 9

CR = 0% Please start pairwise comparison

Calculate

Pairwise Comparison AHP-OS

Evaluation of Criteria for Sustainable Neighborhood

Pairwise Comparison Infrastructure and equipment

28 pairwise comparison(s). Please do the pairwise comparison of all criteria. When completed, click *Check Consistency* to get the priorities.

AHP Scale: 1- Equal Importance, 3- Moderate importance, 5- Strong importance, 7- Very strong importance, 9- Extreme importance (2,4,6,8 values in-between).

With respect to *Infrastructure and equipment*, which criterion is more important, and how much more on a scale 1 to 9?

	A - wrt Infrastructure and equipment - or B?		Equal	How much more?
1	◉ Access to basic infrastructure	○ Disaster response capacity	◉ 1	○2 ○3 ○4 ○5 ○6 ○7 ○8 ○9
2	◉ Access to basic infrastructure	○ Access to public equipment	◉ 1	○2 ○3 ○4 ○5 ○6 ○7 ○8 ○9
3	◉ Access to basic infrastructure	○ Residential garbage management	◉ 1	○2 ○3 ○4 ○5 ○6 ○7 ○8 ○9
4	◉ Access to basic infrastructure	○ Construction disposal management	◉ 1	○2 ○3 ○4 ○5 ○6 ○7 ○8 ○9
5	◉ Access to basic infrastructure	○ Historical infrastructure preservation	◉ 1	○2 ○3 ○4 ○5 ○6 ○7 ○8 ○9
6	◉ Access to basic infrastructure	○ Inclusive design	◉ 1	○2 ○3 ○4 ○5 ○6 ○7 ○8 ○9
7	◉ Access to basic infrastructure	○ Re-used and recycled infrastructure	◉ 1	○2 ○3 ○4 ○5 ○6 ○7 ○8 ○9
8	◉ Disaster response capacity	○ Access to public equipment	◉ 1	○2 ○3 ○4 ○5 ○6 ○7 ○8 ○9
9	◉ Disaster response capacity	○ Residential garbage management	◉ 1	○2 ○3 ○4 ○5 ○6 ○7 ○8 ○9
10	◉ Disaster response capacity	○ Construction disposal management	◉ 1	○2 ○3 ○4 ○5 ○6 ○7 ○8 ○9
11	◉ Disaster response capacity	○ Historical infrastructure preservation	◉ 1	○2 ○3 ○4 ○5 ○6 ○7 ○8 ○9
12	◉ Disaster response capacity	○ Inclusive design	◉ 1	○2 ○3 ○4 ○5 ○6 ○7 ○8 ○9
13	◉ Disaster response capacity	○ Re-used and recycled infrastructure	◉ 1	○2 ○3 ○4 ○5 ○6 ○7 ○8 ○9
14	◉ Access to public equipment	○ Residential garbage management	◉ 1	○2 ○3 ○4 ○5 ○6 ○7 ○8 ○9
15	◉ Access to public equipment	○ Construction disposal management	◉ 1	○2 ○3 ○4 ○5 ○6 ○7 ○8 ○9
16	◉ Access to public equipment	○ Historical infrastructure preservation	◉ 1	○2 ○3 ○4 ○5 ○6 ○7 ○8 ○9
17	◉ Access to public equipment	○ Inclusive design	◉ 1	○2 ○3 ○4 ○5 ○6 ○7 ○8 ○9
18	◉ Access to public equipment	○ Re-used and recycled infrastructure	◉ 1	○2 ○3 ○4 ○5 ○6 ○7 ○8 ○9

19	◉ Residential garbage management	○ Construction disposal management	◉ 1	○ 2 ○ 3 ○ 4 ○ 5 ○ 6 ○ 7 ○ 8 ○ 9
20	◉ Residential garbage management	○ Historical infrastructure preservation	◉ 1	○ 2 ○ 3 ○ 4 ○ 5 ○ 6 ○ 7 ○ 8 ○ 9
21	◉ Residential garbage management	○ Inclusive design	◉ 1	○ 2 ○ 3 ○ 4 ○ 5 ○ 6 ○ 7 ○ 8 ○ 9
22	◉ Residential garbage management	○ Re-used and recycled infrastructure	◉ 1	○ 2 ○ 3 ○ 4 ○ 5 ○ 6 ○ 7 ○ 8 ○ 9
23	◉ Construction disposal management	○ Historical infrastructure preservation	◉ 1	○ 2 ○ 3 ○ 4 ○ 5 ○ 6 ○ 7 ○ 8 ○ 9
24	◉ Construction disposal management	○ Inclusive design	◉ 1	○ 2 ○ 3 ○ 4 ○ 5 ○ 6 ○ 7 ○ 8 ○ 9
25	◉ Construction disposal management	○ Re-used and recycled infrastructure	◉ 1	○ 2 ○ 3 ○ 4 ○ 5 ○ 6 ○ 7 ○ 8 ○ 9
26	◉ Historical infrastructure preservation	○ Inclusive design	◉ 1	○ 2 ○ 3 ○ 4 ○ 5 ○ 6 ○ 7 ○ 8 ○ 9
27	◉ Historical infrastructure preservation	○ Re-used and recycled infrastructure	◉ 1	○ 2 ○ 3 ○ 4 ○ 5 ○ 6 ○ 7 ○ 8 ○ 9
28	◉ Inclusive design	○ Re-used and recycled infrastructure	◉ 1	○ 2 ○ 3 ○ 4 ○ 5 ○ 6 ○ 7 ○ 8 ○ 9

CR = 0% Please start pairwise comparison

Calculate

AHP-OS Latest News

Pairwise Comparison AHP-OS

Evaluation of Criteria for Sustainable Neighborhood

Pairwise Comparison Transport and mobility

10 pairwise comparison(s). Please do the pairwise comparison of all criteria. When completed, click *Check Consistency* to get the priorities.

AHP Scale: 1- Equal Importance, 3- Moderate importance, 5- Strong importance, 7- Very strong importance, 9- Extreme importance (2,4,6,8 values in-between).

With respect to *Transport and mobility*, which criterion is more important, and how much more on a scale 1 to 9?

	A - wrt Transport and mobility - or B?		Equal	How much more?
1	◉ Transportation CO2 emissions	○ Access to public transportation	◉ 1	○2 ○3 ○4 ○5 ○6 ○7 ○8 ○9
2	◉ Transportation CO2 emissions	○ Vehicle and pedestrian traffic	◉ 1	○2 ○3 ○4 ○5 ○6 ○7 ○8 ○9
3	◉ Transportation CO2 emissions	○ Public transportation installations	◉ 1	○2 ○3 ○4 ○5 ○6 ○7 ○8 ○9
4	◉ Transportation CO2 emissions	○ Bicycle installations	◉ 1	○2 ○3 ○4 ○5 ○6 ○7 ○8 ○9
5	◉ Access to public transportation	○ Vehicle and pedestrian traffic	◉ 1	○2 ○3 ○4 ○5 ○6 ○7 ○8 ○9
6	◉ Access to public transportation	○ Public transportation installations	◉ 1	○2 ○3 ○4 ○5 ○6 ○7 ○8 ○9
7	◉ Access to public transportation	○ Bicycle installations	◉ 1	○2 ○3 ○4 ○5 ○6 ○7 ○8 ○9
8	◉ Vehicle and pedestrian traffic	○ Public transportation installations	◉ 1	○2 ○3 ○4 ○5 ○6 ○7 ○8 ○9
9	◉ Vehicle and pedestrian traffic	○ Bicycle installations	◉ 1	○2 ○3 ○4 ○5 ○6 ○7 ○8 ○9
10	◉ Public transportation installations	○ Bicycle installations	◉ 1	○2 ○3 ○4 ○5 ○6 ○7 ○8 ○9

CR = 0% Please start pairwise comparison

Calculate

AHP-OS Latest News

Pairwise Comparison AHP-OS

Evaluation of Criteria for Sustainable Neighborhood

Pairwise Comparison Resources and energy

15 pairwise comparison(s). Please do the pairwise comparison of all criteria. When completed, click *Check Consistency* to get the priorities.

AHP Scale: 1- Equal Importance, 3- Moderate importance, 5- Strong importance, 7- Very strong importance, 9- Extreme importance (2,4,6,8 values in-between).

With respect to *Resources and energy*, which criterion is more important, and how much more on a scale 1 to 9?

	A - wrt Resources and energy - or B?		Equal	How much more?
1	◉ Reduction of water consumption	○ Sewage management	◉ 1	○2 ○3 ○4 ○5 ○6 ○7 ○8 ○9
2	◉ Reduction of water consumption	○ Rainwater management	◉ 1	○2 ○3 ○4 ○5 ○6 ○7 ○8 ○9
3	◉ Reduction of water consumption	○ Energy strategy	◉ 1	○2 ○3 ○4 ○5 ○6 ○7 ○8 ○9
4	◉ Reduction of water consumption	○ Optimization of energetic performance	◉ 1	○2 ○3 ○4 ○5 ○6 ○7 ○8 ○9
5	◉ Reduction of water consumption	○ Low-impact materials	◉ 1	○2 ○3 ○4 ○5 ○6 ○7 ○8 ○9
6	◉ Sewage management	○ Rainwater management	◉ 1	○2 ○3 ○4 ○5 ○6 ○7 ○8 ○9
7	◉ Sewage management	○ Energy strategy	◉ 1	○2 ○3 ○4 ○5 ○6 ○7 ○8 ○9
8	◉ Sewage management	○ Optimization of energetic performance	◉ 1	○2 ○3 ○4 ○5 ○6 ○7 ○8 ○9
9	◉ Sewage management	○ Low-impact materials	◉ 1	○2 ○3 ○4 ○5 ○6 ○7 ○8 ○9
10	◉ Rainwater management	○ Energy strategy	◉ 1	○2 ○3 ○4 ○5 ○6 ○7 ○8 ○9
11	◉ Rainwater management	○ Optimization of energetic performance	◉ 1	○2 ○3 ○4 ○5 ○6 ○7 ○8 ○9
12	◉ Rainwater management	○ Low-impact materials	◉ 1	○2 ○3 ○4 ○5 ○6 ○7 ○8 ○9
13	◉ Energy strategy	○ Optimization of energetic performance	◉ 1	○2 ○3 ○4 ○5 ○6 ○7 ○8 ○9
14	◉ Energy strategy	○ Low-impact materials	◉ 1	○2 ○3 ○4 ○5 ○6 ○7 ○8 ○9
15	◉ Optimization of energetic performance	○ Low-impact materials	◉ 1	○2 ○3 ○4 ○5 ○6 ○7 ○8 ○9
CR = 0% Please start pairwise comparison				
Calculate				

AHP-OS Latest News

Pairwise Comparison AHP-OS

Evaluation of Criteria for Sustainable Neighborhood

Pairwise Comparison Participation and social well-being

3 pairwise comparison(s). Please do the pairwise comparison of all criteria. When completed, click *Check Consistency* to get the priorities.

AHP Scale: 1- Equal Importance, 3- Moderate importance, 5- Strong importance, 7- Very strong importance, 9- Extreme importance (2,4,6,8 values in-between).

With respect to *Participation and social well-being*, which criterion is more important, and how much more on a scale 1 to 9?

	A - wrt Participation and social well-being - or B?		Equal	How much more?
1	◉ Social housing provision	○ Community participation	◉ 1	○2 ○3 ○4 ○5 ○6 ○7 ○8 ○9
2	◉ Social housing provision	○ Neighborhood management	◉ 1	○2 ○3 ○4 ○5 ○6 ○7 ○8 ○9
3	◉ Community participation	○ Neighborhood management	◉ 1	○2 ○3 ○4 ○5 ○6 ○7 ○8 ○9

CR = 0% Please start pairwise comparison

Calculate

AHP-OS Latest News

Pairwise Comparison AHP-OS

Evaluation of Criteria for Sustainable Neighborhood

Pairwise Comparison Neighborhood environment

3 pairwise comparison(s). Please do the pairwise comparison of all criteria. When completed, click *Check Consistency* to get the priorities.

AHP Scale: 1- Equal Importance, 3- Moderate importance, 5- Strong importance, 7- Very strong importance, 9- Extreme importance (2,4,6,8 values in-between).

With respect to *Neighborhood environment*, which criterion is more important, and how much more on a scale 1 to 9?

	A - wrt Neighborhood environment - or B?		Equal	How much more?
1	◉ Heat island	○ Noise pollution	◉ 1	○2 ○3 ○4 ○5 ○6 ○7 ○8 ○9
2	◉ Heat island	○ Light pollution	◉ 1	○2 ○3 ○4 ○5 ○6 ○7 ○8 ○9
3	◉ Noise pollution	○ Light pollution	◉ 1	○2 ○3 ○4 ○5 ○6 ○7 ○8 ○9

CR = 0% Please start pairwise comparison

Calculate
